# Macro- and microstructural assessment of alveolar bone in adults with different vertical facial patterns using cone beam computed tomography

**DOI:** 10.3389/froh.2026.1700017

**Published:** 2026-02-16

**Authors:** Abeer A. Almashraqi, Amal A. Qasem, Aisha M. Yamani, Rahaf T. Alshahrani, Rawan D. Arishi, Maged S. Alhammadi

**Affiliations:** 1 Department of Clinical Oral Health Sciences, College of Dental Medicine, QU Health, Qatar University, Doha, Qatar; 2 General Dentist, College of Dentistry, Jazan University, Jazan, Saudi Arabia; 3 Saudi Board Endodontic Program, Armed Forces Hospital, Southern Region, Saudi Arabia; 4 Department of Oral and Maxillofacial Surgery and Diagnostic Sciences, Najran University, Najran, Saudi Arabia; 5 Orthodontics and Dentofacial Orthopedics, Department of Preventive Dental Sciences, College of Dentistry, Jazan University, Jazan, Saudi Arabia

**Keywords:** bone volume, fractal dimension, histomorphometric, hyperdivergent, inter-radicular bone

## Abstract

**Introduction:**

Vertical facial growth patterns play a crucial role in craniofacial morphology and have significant implications for diagnosis, treatment planning, and long-term stability in orthodontics and maxillofacial surgery. This study sought to comprehensively study the macro- and microstructure of alveolar bone in different vertical facial patterns using cone beam computed tomography (CBCT).

**Methods:**

A cross-sectional study involved 120 CBCT scans divided equally into normodivergent and hyperdivergent vertical facial patterns of both genders. Alveolar bone analyses were conducted for both the maxilla and mandible from central incisors to 2nd molars. Inter-radicular thickness (IRT) measurements were performed at 4 different vertical levels (4, 6, 8 and 11 mm). For the microstructural analysis, the extracted file of area of interest was obtained, and then ImageJ was used to assess trabecular bone thickness (Tb.Th), separation (Tb.SP), bone volume ratio (BV), and fractal dimension (FD). The independent t-test was used to assess the differences across all groups.

**Results:**

Hyperdivergent males exhibited statistically significant thicker IRT, particularly in the posterior maxilla and mandible, with increased Tb.SP, and lower BV in most sites compared to normodivergent facial pattern. In contrast, hyperdivergent females had significantly thinner IRT, specifically in the anterior maxilla and mandible, associated with lower BV (notably between the lateral incisors and canines). Normodivergent males and females showed minimal differences, while the hyperdivergent group demonstrated more pronounced gender-related variation in both macrostructural and microstructural alveolar bone characteristics at different sites.

**Conclusion:**

Hyperdivergent individuals exhibit distinct site- and gender-specific differences at the macrostructural level, accompanied by microarchitectural adaptations across both maxilla and mandible in comparison to normodivergent individuals.

## Introduction

Alveolar bone is crucial for upholding and supporting the teeth in the jaw, keeping the integrity of dental arches, and withstanding the forces of mastication. It is a dynamic structure that remodels in response to functional needs and mechanical stress continuously. The alveolar bone quality and quantity are fundamental for the success of many dental and orthodontic procedures, such as tooth movement during orthodontic treatment, dental implant placement, and maintaining long-term periodontal health (1, 2). Changes in the morphometric and histomorphometric structure of the alveolar bone, such as its thickness, height, and contour, along with the differences in bone density and trabecular microarchitecture, can all significantly influence the treatment outcomes (2). These differences could affect the rate and amount of orthodontic tooth movement, post-treatment stability, and the risk profile of the treatment (dehiscence, fenestration, or root resorption) (3–5).

Bone microarchitecture can be assessed using different established methodologies, including histologic analysis of bone biopsies and micro-computed tomography (micro-CT). Although these techniques provide detailed and direct measurements of trabecular structure, their invasive nature, cost, and limited applicability in routine clinical settings restrict their widespread use. Image-based analytical approaches have therefore gained increasing attention as practical alternatives for evaluating bone microarchitecture. Among these, trabecular, fractal dimension (FD), and bone volume ratio (BV) analysis are mathematical image-analysis techniques that quantify the complexity and spatial organization of trabecular bone patterns. Previous studies have demonstrated significant correlations between fractal dimension and conventional histomorphometric and micro-CT parameters, supporting its use as a surrogate indicator of bone microarchitecture (6, 7).

Vertical facial growth patterns (VFGP) play a vital role in craniofacial morphology, which affects diagnosis, treatment planning, and long-term stability in orthodontics and maxillofacial surgery (8). One notable pattern is the hyperdivergent individuals, who are usually identified by increased lower anterior facial height, steep mandibular plane angle, and a tendency toward skeletal open bite. Remarkably, some previous studies suggested that the muscles of mastication are overstretched in hyperdivergent individuals, which influences their strength and therefore negatively impacts the quality of the alveolar bone (9, 10). On that account, understanding the characteristics of alveolar bone in different VFGP is important for planning safe and effective dental and orthodontic interventions.

Remarkably, different research has been conducted to assess the alveolar bone in different VFGP, where most of them focused on macrostructural assessment, particularly the cortical bone thickness (11–15), along with the assessment of bone density (14, 16–18). Their results demonstrated great variation, and most of them studied specific regions in the jaw only. On the other hand, a few studies have focused on the histomorphometric analysis, specifically FD, which was also associated with inconsistent findings across the research (11, 19). Notably, although macrostructural assessment and bone density have been reported in various vertical facial individuals, the complexity and space-filling nature of their trabecular architecture remains not fully explored. Moreover, little is known about how external alveolar dimensions correlate with internal bone quality across different VFGP. The qualitative and quantitative evaluation of this complex area may guide the clinician, implantologist, or orthodontist to take specific anatomical factors into account before planning dental implants or biomechanical aspects before developing orthodontic treatment, ultimately enhancing the outcome results. Therefore, this study aimed to comprehensively assess the inter-radicular bone thickness (IRT) dimensions and trabecular bone microarchitecture in hyperdivergent individuals compared to normodivergent individuals in the maxilla and mandible across both genders.

## Materials and methods

### Study design and ethical considerations

This is a cross-sectional comparative study that was conducted in compliance with the Helsinki Convention (20) and was approved by the Research Ethics Committee (REC42/1/049) at the College of Dentistry, Jazan University, Saudi Arabia. Informed consent was obtained from all subjects during the registration process.

### Sample size

The Sample size was calculated with an alpha value of 0.05 and a power of 85% based on the study conducted by Ono et al. (21) in which the mean and standard deviation of the cancellous bone thickness in the first premolar region in adult females were 7.03 ± 1.33 and 5.66 ± 2.01 mm in the low-angle and high-angle subjects, respectively. The calculation showed a minimum sample of 29 subjects required in this study in each group.

### Inclusion and exclusion criteria

The sampling was performed retrospectively on participants who visited the oral radiology department and were referred from other departments for a full-head CBCT. Subjects were randomly selected after applying the predefined inclusion and exclusion criteria. The participants who fulfilled the following inclusion criteria: 1) aged 18–40 years; 2) all permanent teeth present except for the third molars; 3) skeletal Class I based on ANB angle; 4) high-quality scans obtained under consistent settings; and 5) complete dental and medical history were recruited for the current study. However, the patients who had 1) syndromes or bone diseases; 2) a history of facial trauma or maxillofacial surgery; and 3) prior dentoalveolar surgical, periodontal, or orthodontic treatment were excluded from the study. An expert oral and maxillofacial radiologist assessed the CBCT scans for any undetected pathologies (A.A.).

### CBCT scanning

CBCT images were captured using the i-Cat CBCT machine (Imaging Sciences International, Hatfield, PA, USA). The distance from the source to the detector was set at 67.5 cm. A voxel dimension of 0.3 mm and a slice thickness of 2 mm were selected with settings of 120 kV, 18.54 mAs, and an exposure time of 8.9 s. The field of view used was 23 × 17 cm. Then, CBCT scans of 120 patients who fulfilled the selection criteria were retrieved and divided based on the mandibular plane angle (MPA) into four equal groups (30 patients each) of normo- and hyperdivergent VFGP of both genders.

### CBCT assessments

All CBCT images were assessed for alveolar bone macrostructural features by two authors (A.Q., R.A.) and for microstructural architecture by two other authors (A.Y., R.T.A.). Additionally, one oral and maxillofacial radiologist (A.A.), with over 15 years of experience in interpreting CBCT images, supervised both macro- and micro-structural analyses. All assessors were blinded to participants' details and grouping. To ensure consistency, intra- and inter-examiner reliability analyses were conducted on 20% of the selected sample.

The intra- and inter-examiner reliability demonstrated a high level of agreement. For IRT measurement, intra- and inter-examiner reliabilities ICC values ranged from the lowest value of 0.765 [95% confidence interval (CI), 0.407–0.907] between lateral incisor and canine, and the highest value of 0.963 (95% CI, 0.908–0.986) between 2nd premolar and 1st molar.

### Macrostructural assessment

**Figure 1:**
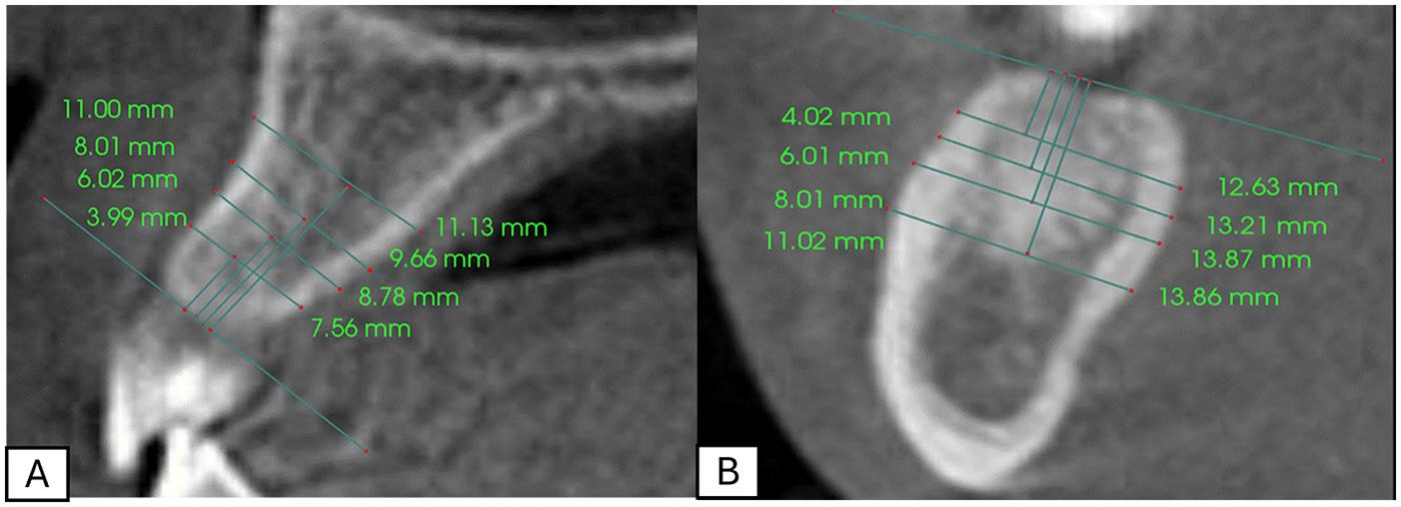
Inter-radicular measurements at 4, 6, 8, and 11 mm levels in **(A)** maxilla and **(B)** mandible.

Following the proper orientation of the multiplanar views in the three orthogonal planes, a full set of inter-radicular bone thicknesses at 4, 6, 8, and 11 mm in the maxillary inter-radicular areas mesial to the second molars was measured using Anatomage 6.03 software (Anatomage, San Jose, CA). To standardize the measurements, a tangent line was drawn along the alveolar crest, followed by a perpendicular line to define the measurement points at 4, 6, 8, and 11 mm. Finally, the inter-radicular bone thickness was measured from the buccal to lingual side, parallel to the tangent plane, as illustrated in Figure 1.

### Microstructural assessment

**Figure 2:**
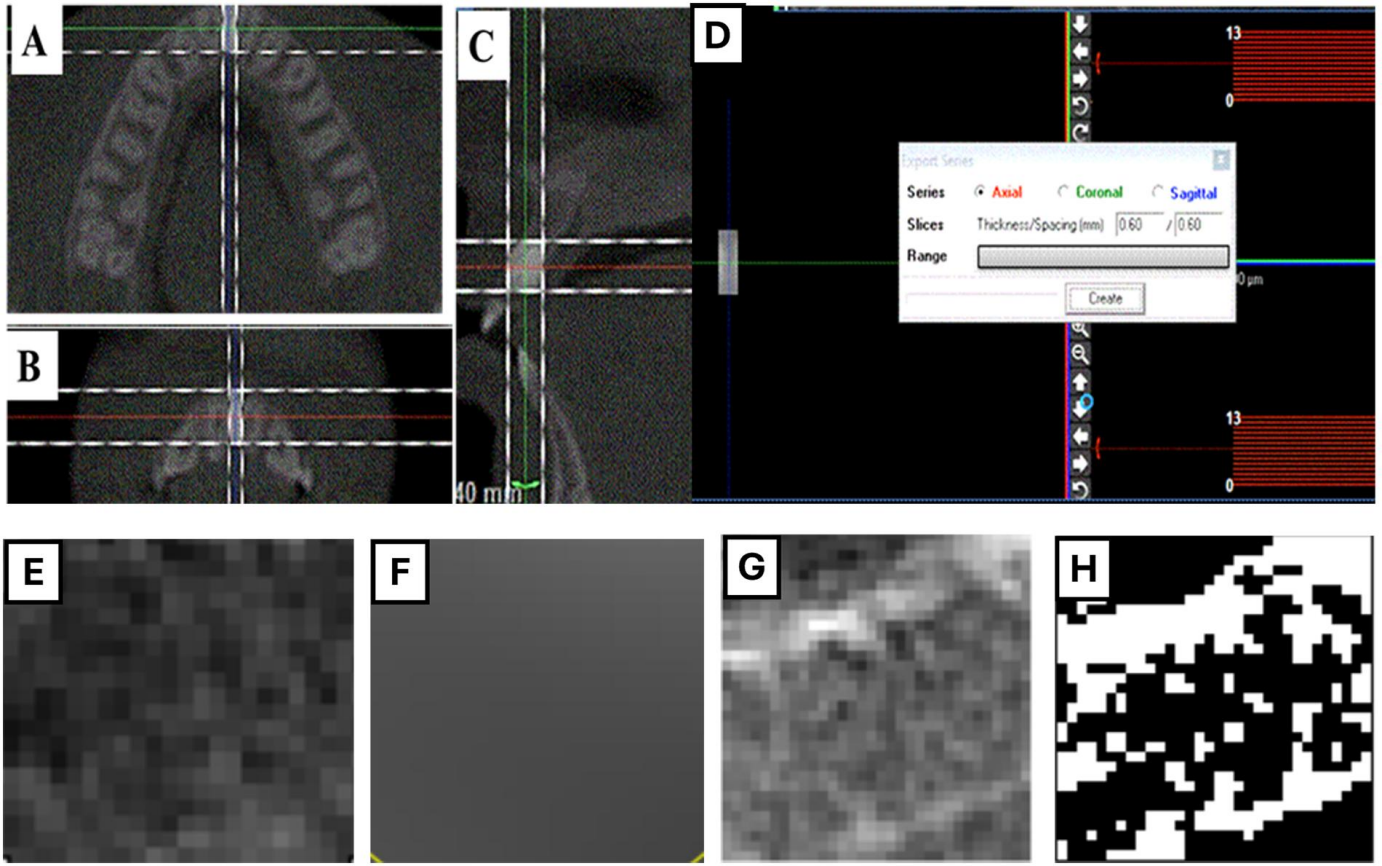
Microstructural analysis, **(A–D)** extraction of the DICOM file of the region of interest. **(E–H)** Histomorphometric analysis using ImageJ with **(E)** Image of region of interest, **(F)** Image of Gaussian blur, **(G)** Subtracted image, and **(H)** Converted to a binary image.

First, bone cuboids from CBCT scans were extracted for each inter-radicular area mesial to the second molars using WhiteFox imaging software version 3 (Acteon Group, Milan, Italy). The “Trim Image” tool was used to obtain the largest cuboid from each inter-radicular bone of all teeth (Figure 2). The “Export Series” tool was then employed to save the axial slices of the cuboid as Digital Imaging and Communications in Medicine (DICOM) files (Figure 2). These DICOM files were later used for bone histomorphometric analysis, which was performed using Fiji software (a special version of ImageJ) with the BoneJ2 plugin (W.S. Rasband, ImageJ; US National Institutes of Health, Bethesda, MD).

The measurements of trabecular microstructure followed the method described by Barngkgei et al. (22), Almashraqi et al. (23), and Carvalho et al. (24). The DICOM file of the region of interest was imported as an image sequence, duplicated, filtered using a Gaussian blur, subtracted, and converted to a binary (8-bit) image (Figure 2). Then, trabecular bone thickness (Tb Th) and trabecular bone separation (Tb Sp) were measured using the “Thickness” tool from the BoneJ dropdown menu. Additionally, bone volume ratio and fractal dimensions were calculated using the “Fraction, Area/Volume Fraction” and “Fractal Dimension” tools, respectively, from the BoneJ dropdown menu. All measurements were reported as mean values with standard deviations.

### Statistical analysis

Data was inputted and analyzed using the Statistical Package for Social Sciences (SPSS) software, Version 21 (IBM Corp., Armonk, NY). Intra-class correlation coefficients (ICCs) were computed to evaluate the reproducibility of the measurements. Normal distribution of the data was assessed using the Kolmogorov–Smirnov test. For all measurements, the average of both sides was calculated and analyzed. Descriptive statistics, including means and standard deviations, were calculated and presented. All comparisons of micro- and macrostructural analysis across different groups were conducted using an independent t-test. A *P* value of ≤0.05 was considered significant.

## Results

A total of 120 CBCT scans were assessed, divided equally into 30 patients in normo-divergent and hyperdivergent groups across both genders. The demographic data of the selected sample is presented in Table 1.

**Table 1:** General characteristics of participants.

Parameters	Gender	Normo-divergent	Hyper-divergent
Number of participants	Male	30	30
	Female	30	30
Age Mean (SD)	Male	22.6 (6.5)	22.21 (5.2)
	Female	20 (7.4)	22.48 (6.63)
ANB Mean (SD)	Male	1.77 (1.19)	2.47 (0.63)
	Female	2.26 (1.22)	2.27 (1.2)
SN-MP Mean (SD)	Male	31.47° (2.87)	40.94° (4.51)
	Female	30.59° (2.22)	43.73° (2.44)

### Inter-radicular bone thickness measurements

**Figure 3:**
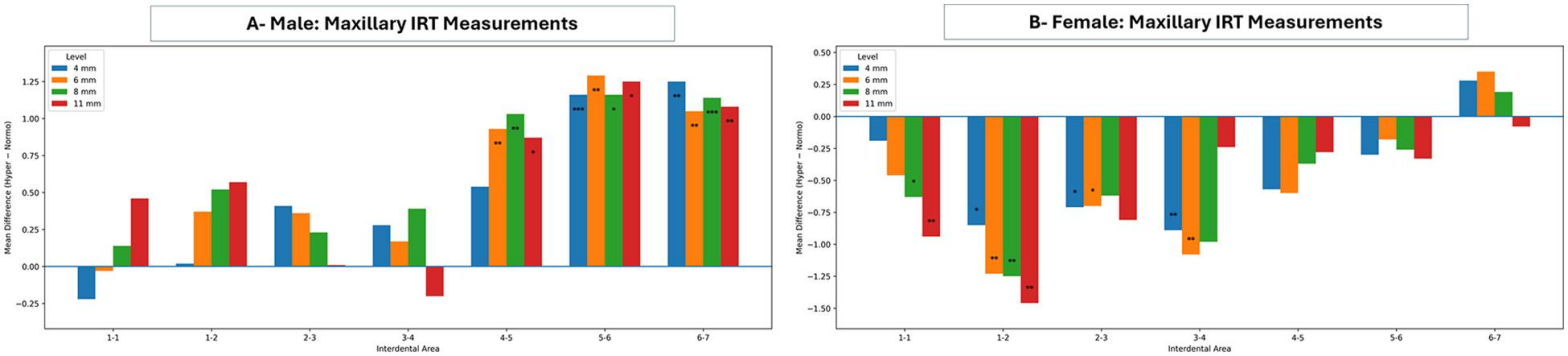
Maxillary inter-radicular mean differences (hyperdivergent-normodivergent) across inter-radicular areas at 4, 6, 8, and 11 mm in males **(A)** and females **(B)** positive values indicate greater inter-radicular dimensions in the hyperdivergent group, whereas negative values indicate greater dimensions in the normodivergent group. Statistical significance is denoted by asterisks (*p < 0.05, **p < 0.01, ***p < 0.001); the horizontal line at zero indicates no difference between groups.

As presented in Figure 3, the comparison of the IRT measurements in different VFGP showed that the hyperdivergent males exhibited significantly thicker IRT at posterior maxillary sites at all levels (from distal of the 1st premolar to the mesial of the 2nd molar) compared to normodivergent males. Conversely, hyperdivergent females revealed significantly thinner IRT at anterior maxillary sites, specifically at the 4 and the 6 mm levels (from mesial of the central incisor to the mesial of the 1st premolar) compared to normodivergent females. The comparison of maxillary IRT measurements between male and female groups in normo-divergent facial type showed no statistically significant differences. However, the same comparison in the hyper-divergent facial type revealed statistically significant differences at most sites and levels, with a pattern of thicker bone in the male group compared to the female group (Supplementary Table S1).

**Figure 4:**
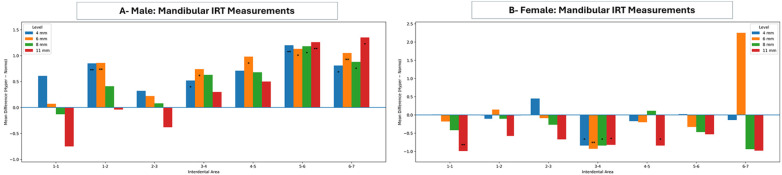
Mandibular inter-radicular mean differences (hyperdivergent-normodivergent) across inter-radicular areas at 4, 6, 8, and 11 mm in males **(A)** and females **(B)** positive values indicate greater inter-radicular dimensions in the hyperdivergent group, whereas negative values indicate greater dimensions in the normodivergent group. Statistical significance is denoted by asterisks (*p < 0.05, **p < 0.01, ***p < 0.001); the horizontal line at zero indicates no difference between groups.

Similar to the maxilla, the comparison between normodivergent and hyperdivergent groups in the mandibular IRT showed thicker and thinner alveolar bone in hyperdivergent males and hyperdivergent females, respectively, compared to their matched normodivergent groups (Figure 4). Further comparisons of mandibular IRT measurements were conducted between male and female groups in different VFGP, showing no statistically significant differences at all sites, except for thicker bone between canines and 1st premolars at the 4 and the 6 mm levels in normo-divergent females compared to the male group. In contrast, hyperdivergent females showed thinner alveolar bone than males, with statistically significant differences at different sites and levels (e.g., between central incisors and central incisors and lateral incisors at the 4 mm level, between canines and 1st premolars at the 6 mm and the 8 mm levels, and between 2nd premolars and 1st molars at all levels) (Supplementary Table S2).

### Microstructural analysis

**Figure 5:**
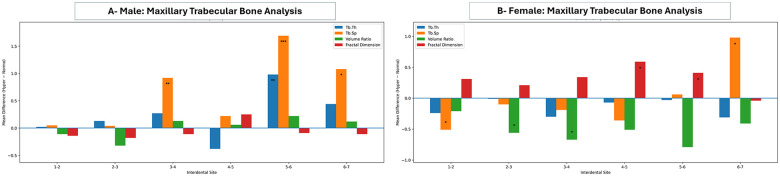
Mean differences (hyperdivergent-normodivergent) in maxillary trabecular bone parameters across interdental sites (1–2 to 6–7) in males **(A)** and females **(B)**. Bar charts depict trabecular thickness (Tb.Th), trabecular separation (Tb.Sp), volume ratio, and fractal dimension. Positive values indicate higher measurements in the hyperdivergent group, whereas negative values indicate higher measurements in the normodivergent group. Statistical significance is indicated by asterisks (*p < 0.05, **p < 0.01, ***p < 0.001); the horizontal line at zero denotes no difference between groups.

Maxillary trabecular bone measurements in male and female groups of normo- and hyper-divergent VFGP are presented in Figure 5. Most sites showed no statistically significant differences between normo- and hyper-divergent male groups across most variables. Even though it is worth mentioning that site-difference findings were observed. Hyperdivergent females showed a lower BV (between lateral incisors and canines and between canines and 1st premolars; *p*-value of 0.033 and 0.031, respectively), a higher FD (between 1st and 2nd premolars and between 2nd premolars and 1st molars; *p*-value of 0.0342), and a decrease Tb SP in all sites (between central and lateral incisors; *p*-value of 0.017) except an increase Tb SP between 1st and 2nd molars; *p*-value of 0.038.

Additionally, there were no statistically significant differences between males and females in the normo-divergent group, except for a higher BV between canines and 1st premolars and between 2nd premolars and 1st molars, as well as increased Tb SP between 2nd premolars and 1st molars in females compared to males. On the other hand, the hyper-divergent group showed more statistically significant differences between males and females compared to the normo-divergent group. These differences were mainly involved decreased Tb Th and Tb Sp in females compared to males, specifically between lateral incisors and canines, canines and1st premolars, and between 2nd premolars and 1st molars (Supplementary Table S3).

**Figure 6:**
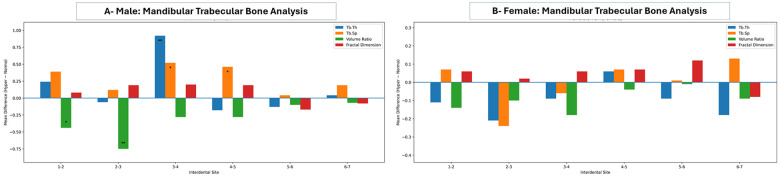
Mean differences (hyperdivergent-normodivergent) in mandibular trabecular bone parameters across interdental sites (1–2 to 6–7) in males **(A)** and females **(B)**. Bar charts depict trabecular thickness (Tb.Th), trabecular separation (Tb.Sp), volume ratio, and fractal dimension. Positive values indicate higher measurements in the hyperdivergent group, whereas negative values indicate higher measurements in the normodivergent group. Statistical significance is indicated by asterisks (*p < 0.05, **p < 0.01, ***p < 0.001); the horizontal line at zero denotes no difference between groups.

Regarding the analysis of mandibular trabecular bone, comparisons between normo- and hyper-divergent VFGP across both genders are presented in Figure 6. The hyper-divergent group in males revealed a lower BV and an increased Tb SP compared to the normo-divergent group. Of these, statistically significant differences in BV were found between the central and lateral incisors and between the lateral incisor and canines, while significant differences in Tb SP were detected between the canines and 1st premolars and the 1st premolars and 2nd premolars. Additionally, Tb Th was increased in the hyper-divergent group when compared to the normo-divergent group in the anterior mandible, with a statistically significant difference between the canines and 1st premolars.

In contrast to the maxilla, the trabecular bone analysis of hyper-divergent facial type showed no statistically significant differences between male and female groups. For the normo-divergent individuals, the Tb Th in the female was significantly increased between the central and lateral incisors and between canines and 1st premolars, along with a significant increase in Tb SP between canines and 1st premolars and between 1st and 2nd molars in comparison with the male group (Supplementary Table S4).

## Discussion

Alveolar bone morphology is a fundamental factor in periodontal health, implant placement, and orthodontic treatment. Based on a previous theory, different vertical facial growth patterns influence the quality and quantity of alveolar bone, with hyperdivergent individuals having weaker muscles and, consequently, reduced bone quality and quantity (9, 10). The present study assessed the alveolar bone microarchitecture and macrostructure in different VFGP, specifically in normodivergent and hyperdivergent individuals across both genders, using CBCT images. The results showed that hyperdivergent males have thicker IRT mainly in the posterior maxilla and mandible compared to normodivergent males, associated with a lower BV, inconsistent FD patterns, and a significant increase in the Tb SP. In contrast, hyperdivergent females showed overall thinner IRT, particularly in the anterior maxilla and a few sites in the mandible, associated with a lower BV, a higher FD, and a lesser Tb SP in the maxilla, compared to normodivergent females. Remarkably, hyperdivergent males had thicker IRT compared to the female group, whereas no noticeable differences were found between both genders in normodivergent facial pattern. The results of the current study support the above-mentioned theory and reveal distinct, site- and gender-specific differences, highlighting the complexity of skeletal adaptation in different VFGP, which have clinical implications for orthodontic and dental implant planning.

The inter-radicular bone thickness dimension findings of hyperdivergent females demonstrated consistently thinner alveolar bone, particularly significant in the anterior maxillary and mandibular regions, compared to normodivergent females. This is consistent with the previous studies that reported a thinner alveolus in the hyperdivergent facial pattern (19, 25–27). However, the present study found an opposite finding in the hyperdivergent males, which contradicts those of the previously mentioned studies. These discrepancies could be attributed to the fact that most previous research didn't stratify their assessments by gender, included a mixed sample, compared hyperdivergent with hypodivergent individuals rather than normodivergent, or differed in age range, method of measurements, and skeletal Classes. Remarkably, this study highlights that gender-specific variations in the assessment of the alveolar bone have been overlooked in previous reports.

On the other hand, some other studies, as the study conducted by Casanova-Sarmiento et al. (12) and Akbulut and Bayrak (11) reported that both normodivergent and hyperdivergent individuals have thinner alveolar bone than hypodivergent individuals. Additionally, Horner et al. (15) reported that hyperdivergent individuals exhibited thinner cortical bone in some sites without significant changes in the cancellous bone compared to hypodivergent individuals. Similar to the findings of the present study, Hoang et al. (28) observed a thicker alveolus in the anterior mandible at the level of the apex of the root and alveolar crest in hyperdivergent patients. Furthermore, while other research found lower bone density in hyperdivergent individuals compared to normodivergent and hypodivergent groups, others reported the opposite (13, 29). This highlights the considerable variation in the literature on this topic and underscores the need for further studies that consider all factors potentially contributing to alveolar bone differences.

Even though the hyperdivergent male showed an increase in the IRT dimension, this does not necessarily indicate a high bone quality, as the bone microarchitecture needs to be assessed as well. Notably, the hyperdivergent male showed a lower BV in the mandible and anterior maxilla, along with increased Tb SP in different sites, particularly the posterior maxilla, compared to the normodivergent male. These findings are consistent with Hasani et al. (14) who found that the hyperdivergent individuals exhibited less cortical bone density posteriorly. Similarly, the hyperdivergent females showed in the present study a lower BV in both the maxilla and mandible in comparison to the normodivergent females. These findings reflect the presence of reduced bone mass and density in the hyperdivergent individuals of both genders. Consistent with the findings of other studies that reported a lower bone density in the hyperdivergent individuals, such as the study conducted by Ozdemir et al. (17), who reported that the hyperdivergent individuals have unfavorable cortical density on the maxillary buccal side. Moreover, Li et al. (16) found that hyperdivergent individuals exhibited significantly lower alveolar bone density at the tested miniscrew sites compared to normodivergent and hypodivergent individuals.

It is worth mentioning that FD was not statistically significant across all groups, except for higher FD between the 1st and 2nd premolars and the 2nd premolar and 1st molar in the hyperdivergent female group compared to the normodivergent female group. This finding is consistent with the study conducted by Akbulut and Bayrak et al. (11), who reported that the hyperdivergent individuals showed no statistically significant differences in the bone complexity at the anterior mandibular alveolus compared to normodivergent and hypodivergent groups. Conversely, Gonca et al. (19) found statistically significant differences in FD of the hyperdivergent group when compared to the normodivergent and hypodivergent groups. This discrepancy could be ascribed to the differences in the assessment methods, as they based their analysis on panoramic radiographs, whereas the present study used CBCT.

Interestingly, although the FD was not statistically significant at most sites across all groups, it exhibited different patterns that underscore the need to interpret all microstructural changes collectively for a better understanding. It is well known that a high FD is accompanied by increased structural complexity, and vice versa (18). Remarkably, the lower BV in the hyperdivergent groups was associated, at most sites, with a higher FD, particularly in females (both arches) and in males (mandible). This suggests a compensatory trabecular arrangement in response to the lower bone mass, which aligns with the concept of Wolff's law that proposed a bone adaptation to mechanical demands by reorganizing its internal architecture even when overall bone volume is limited (30). Even though a higher FD was found, an increase in Tb SP was also observed along with the lower BV, suggesting the presence of low bone mass and less interconnected trabeculae.

On the other hand, a few sites in the anterior maxilla and posterior mandible of the hyperdivergent male group and posterior maxilla and mandible (between 1st and 2nd molars) of the hyperdivergent female group exhibited both a lower BV and FD, along with comparable Tb Th and Tb SP, compared to normodivergent. This indicates a reduced bone mass and a lack of structural complexity. These regions may represent weak areas that require special attention during orthodontic or surgical treatment. Conversely, one site (between 1st and 2nd premolars) in the maxilla of hyperdivergent male individuals showed both a higher BV and FD compared to the normodivergent male group, suggesting a high bone mass and complex structure in this region.

Additionally, statistically significant gender differences further highlight the changes accompanied by different VFGP. Specifically, hyperdivergent males showed thicker IRT, particularly in the maxilla and posterior mandible, compared to hyperdivergent females. Conversely, no significant differences were reported between the male and female groups in microstructural analysis, except at a few sites across both vertical growth patterns. Unfortunately, no research directly comparing these macro- and microstructural gender differences could be identified; however, some studies on bone density and cortical bone thickness provide a basis for comparison. The findings of the microstructural analysis of the present study are consistent with studies that assessed cortical bone density in different VFGP, which reported no statistically significant differences between genders across all groups (14, 31, 32). In contrast, other studies found that females exhibited higher cortical bone density than male individuals (13, 29, 33). On the other hand, some studies reported no statistically significant differences between males and females in cortical bone thickness (19, 32, 34, 35). These differences may be attributed to the differences in the parameters assessed and the methods of evaluation.

From a clinical standpoint, the present findings highlight the importance of accounting for VFGP and gender differences during treatment planning. In orthodontics, regions exhibiting higher IRT may allow effective use of cortical anchorage during tooth movement, whereas caution is warranted in thinner areas to reduce the risk of bone fenestration. Furthermore, in sites demonstrating a marked increase in Tb.Sp, reinforcement of anchorage is recommended to minimize the risk of space loss during maximum incisor retraction. From an implantology perspective, both IRT and Tb.Sp should be carefully considered during implant planning. In areas characterized by lower IRT and greater Tb.Th, the use of longer implants may be advantageous to compensate for the reduced radicular distance. Conversely, in regions with higher IRT and increased Tb.Sp, shorter and wider implants may provide improved primary stability. Collectively, these considerations underscore the clinical relevance of individualized treatment planning based on underlying bone microstructural characteristics.

While this study provided valuable and comprehensive macro- and microstructural analysis, along with site- and gender-specific insights using three-dimensional imaging, its limitations include the absence of a hypodivergent group for comparison. Additionally, measuring cortical bone thickness, bone density, and correlating them with the microstructural findings can offer another valuable dimension to the assessment. Moreover, the results of the present study cannot be generalized, as it was a cross-sectional study, conducted in a specific population, and a particular age group. Further studies that account for all these factors would help further elucidate the dynamic nature of bone adaptation in different facial pattern groups.

## Conclusion

Hyperdivergent individuals exhibit distinct site- and gender-specific differences at the macrostructural level, accompanied by microarchitectural adaptations across both maxilla and mandible in comparison to normodivergent individuals. Hyperdivergent males tend to have thicker IRT but a lower BV and an increase in Tb SP, while hyperdivergent females show thinner IRT with a lower BV overall, compared to the normodivergent individuals. Specific sites in the hyperdivergent individuals, particularly the anterior maxilla and posterior mandible in males and the area between the 1st and 2nd molars in the maxilla and mandible in females, displayed both low bone mass and structural complexity. Conversely, the region between the 1st and 2nd premolars demonstrated both high bone mass and complexity, reflecting good bone quality with satisfactory bone adaptation at this site.

The results of the present study emphasize the importance of individualized skeletal assessment, incorporating both macro- and microstructure analyses, as part of orthodontic and surgical planning. A prospective clinical trial considering all the mentioned variables with a thorough investigation of the rate of tooth movement and the overall treatment time, and correlating the results to the macro- and micro-structural elements of the selected sample. This concept needs to be applied to the dental implant longitudinal assessment of success and failure rates related to the abovementioned categories.

## Data Availability

The raw data supporting the conclusions of this article will be made available by the authors, without undue reservation.
