# Supplementary material for: Macro- and microstructural assessment of alveolar bone in adults with different vertical facial patterns using cone beam computed tomography
Source: Front Oral Health. 2026 Feb 16;7:1700017. doi: 10.3389/froh.2026.1700017 (PMC12950796; doi:10.3389/froh.2026.1700017)
Supplement: Supplementary file 1 [file Table1.docx]

Supplementary Table 1: Comparative statistical analysis of the maxillary inter-radicular measurements between normo- and hyper-divergent facial types in male and female groups using an independent t-test

| **Area** | **Level (mm)** | **Male** | | | ***Female*** | | | ***P* value** | |
| --- | --- | --- | --- | --- | --- | --- | --- | --- | --- |
|  |  | **Normo-divergent**  **Mean (SD)** | **Hyper-Divergent**  **Mean (SD)** | ***P* value** | **Normo-divergent**  **Mean (SD)** | **Hyper-Divergent**  **Mean (SD)** | ***P* value** | **Normo-divergent**  **Male/Female** | **Hyper-divergent**  **Male/Female** |
| **1-1** | **4** | 6.45 (0.71) | 6.23 (0.59) | 0.303 | 6.17 (0.79) | 5.98 (0.67) | 0.43 | 0.249 | 0.23 |
|  | **6** | 6.59 (0.70) | 6.56 (0.80) | 0.922 | 6.33 (1.01) | 5.87 (0.69) | 0.102 | 0.357 | 0.006^**^ |
|  | **8** | 6.62 (1.13) | 6.76 (0.95) | 0.674 | 6.22 (0.87) | 5.59 (0.89) | 0.032^*^ | 0.218 | 0.001^***^ |
|  | **11** | 7.01 (1.14) | 7.47 (1.41) | 0.269 | 6.63 (0.72) | 5.69 (1.26) | 0.007^**^ | 0.22 | 0.001^***^ |
| **1-2** | **4** | 6.87 (0.80) | 6.89 (0.59) | 0.934 | 7.46 (1.27) | 6.61 (1.04) | 0.028^*^ | 0.089 | 0.316 |
|  | **6** | 7.16 (1.07) | 7.53 (0.58) | 0.187 | 7.87 (1.28) | 6.64 (1.22) | 0.005^**^ | 0.083 | 0.006^**^ |
|  | **8** | 7.35 (1.24) | 7.87 (0.70) | 0.119 | 7.88 (1.35) | 6.63 (1.28) | 0.005^**^ | 0.211 | 0.001^***^ |
|  | **11** | 7.78 (1.14) | 8.35 (0.80) | 0.076 | 8.47 (1.62) | 7.01 (1.50) | 0.005^**^ | 0.13 | 0.001^***^ |
| **2-3** | **4** | 7.38 (0.80) | 7.79 (0.79) | 0.109 | 7.54 (.69) | 6.83 (0.93) | 0.01^*^ | 0.505 | 0.001^***^ |
|  | **6** | 7.81 (1.05) | 8.17 (0.80) | 0.232 | 7.85 (0.84) | 7.15 (1.09) | 0.031^*^ | 0.889 | 0.002^**^ |
|  | **8** | 7.90 (0.97) | 8.13 (1.03) | 0.478 | 7.66 (0.96) | 7.04 (1.27) | 0.094 | 0.427 | 0.005^**^ |
|  | **11** | 8.15 (1.15) | 8.16 (1.29) | 0.977 | 8.00 (1.55) | 7.19 (1.49) | 0.1 | 0.727 | 0.034^*^ |
| **3-4** | **4** | 9.04 (0.77) | 9.32 (0.68) | 0.233 | 9.26 (0.94) | 8.37 (0.94) | 0.005^**^ | 0.442 | 0.001^***^ |
|  | **6** | 9.43 (1.24) | 9.60 (0.73) | 0.591 | 9.69 (1.13) | 8.61 (1.31) | 0.008^**^ | 0.487 | 0.006^**^ |
|  | **8** | 9.42 (1.68) | 9.81 (0.97) | 0.379 | 10.01 (1.43) | 9.03 (1.97) | 0.079 | 0.239 | 0.122 |
|  | **11** | 10.32 (1.97) | 10.12 1(.130 | 0.687 | 10.79 (2.19) | 10.55 (3.73) | 0.807 | 0.486 | 0.624 |
| **4-5** | **4** | 9.99 (0.83) | 10.53 (0.96) | 0.066 | 10.16 (1.03) | 9.59 (0.98) | 0.083 | 0.574 | 0.004^**^ |
|  | **6** | 9.71 (0.99) | 10.64 (0.71) | 0.002^**^ | 9.91 (1.07) | 9.31 (1.02) | 0.075 | 0.533 | 0.001^***^ |
|  | **8** | 9.48 (1.14) | 10.51 (0.71) | 0.002^**^ | 9.68 (1.31) | 9.31 (1.51) | 0.409 | 0.607 | 0.003^**^ |
|  | **11** | 9.45 (1.66) | 10.32 (0.95) | 0.049^*^ | 10.30 (1.63) | 10.02 (1.91) | 0.615 | 0.108 | 0.527 |
| **5-6** | **4** | 11.58 (1.07) | 12.74 (1.04) | 0.001^***^ | 11.44 (1.43) | 11.14 (1.00) | 0.456 | 0.717 | 0.001^***^ |
|  | **6** | 11.48 (1.33) | 12.77 (1.11) | 0.002^**^ | 11.21 (1.56) | 11.03 (1.05) | 0.676 | 0.558 | 0.001^***^ |
|  | **8** | 11.55 (1.65) | 12.71 (1.35) | 0.02^*^ | 11.38 (1.49) | 11.12 (1.15) | 0.544 | 0.728 | 0.001^***^ |
|  | **11** | 11.60 (2.02) | 12.85 (1.71) | 0.042^*^ | 12.30 (1.47) | 11.97 (1.28) | 0.454 | 0.22 | 0.074 |
| **6-7** | **4** | 13.97 (1.36) | 15.22 (1.08) | 0.003^**^ | 13.28 (1.46) | 13.56 (1.13) | 0.516 | 0.132 | 0.001^***^ |
|  | **6** | 14.45 (1.29) | 15.50 (1.02) | 0.007^**^ | 13.57 (1.55) | 13.92 (0.88) | 0.388 | 0.059 | 0.001^***^ |
|  | **8** | 14.18 (1.14) | 15.32 (0.88) | 0.001^***^ | 13.97 (1.77) | 14.16 (0.87) | 0.66 | 0.651 | 0.001^***^ |
|  | **11** | 14.52 (1.03) | 15.60 (1.17) | 0.004^**^ | 14.71 (1.99) | 14.63 (0.69) | 0.869 | 0.701 | 0.004^**^ |

*^*, **, ***:^ P-value*
