# Supplementary material for: Macro- and microstructural assessment of alveolar bone in adults with different vertical facial patterns using cone beam computed tomography
Source: Front Oral Health. 2026 Feb 16;7:1700017. doi: 10.3389/froh.2026.1700017 (PMC12950796; doi:10.3389/froh.2026.1700017)
Supplement: Supplementary file 2 [file Table2.docx]

Supplementary Table 2: Comparative statistical analysis of the mandibular inter-radicular measurements between normo- and hyper-divergent facial types in male and female groups using an independent t-test

| **Area** | **Level (mm)** | **Male** | | | ***Female*** | | | ***P* value** | |
| --- | --- | --- | --- | --- | --- | --- | --- | --- | --- |
|  |  | **Normo-divergent**  **Mean (SD)** | **Hyper-Divergent**  **Mean (SD)** | ***P* value** | **Normo-divergent**  **Mean (SD)** | **Hyper-Divergent**  **Mean (SD)** | ***P* value** | **Normo-divergent**  **Male/Female** | **Hyper-divergent**  **Male/Female** |
| **1-1** | **4** | 5.46 (1.01) | 6.07 (0.90) | 0.052 | 5.27 (0.28) | 5.28 (0.85) | 0.984 | 0.436 | 0.007^**^ |
|  | **6** | 5.65 (0.84) | 5.72 (0.90) | 0.782 | 5.48 (0.60) | 5.30 (0.81) | 0.427 | 0.468 | 0.123 |
|  | **8** | 5.77 (1.05) | 5.64 (1.03) | 0.702 | 5.91 (0.42) | 5.49 (1.04) | 0.109 | 0.582 | 0.655 |
|  | **11** | 6.72 (1.44) | 5.97 (1.07) | 0.072 | 7.28 (0.89) | 6.29 (1.15) | 0.004^**^ | 0.149 | 0.375 |
| **1-2** | **4** | 5.82 (0.68) | 6.67 (1.01) | 0.004^**^ | 6.00 (0.39) | 5.89 (0.92) | 0.649 | 0.33 | 0.015^*^ |
|  | **6** | 5.63 (0.65) | 6.49 (1.07) | 0.004^**^ | 5.71 (0.48) | 5.85 (1.20) | 0.642 | 0.655 | 0.081 |
|  | **8** | 5.78 (0.70) | 6.19 (1.06) | 0.16 | 6.06 (0.56) | 5.95 (1.18) | 0.687 | 0.169 | 0.492 |
|  | **11** | 6.57 (0.81) | 6.53 (1.05) | 0.884 | 7.06 (1.02) | 6.50 (1.13) | 0.103 | 0.098 | 0.934 |
| **2-3** | **4** | 6.49 (0.74) | 6.81 (0.83) | 0.212 | 6.45 (0.70) | 6.90 (1.40) | 0.206 | 0.847 | 0.797 |
|  | **6** | 6.46 (0.81) | 6.68 (0.87) | 0.397 | 6.36 (0.85) | 6.27 (1.31) | 0.805 | 0.717 | 0.252 |
|  | **8** | 6.48 (0.84) | 6.56 (1.07) | 0.804 | 6.51 (0.93) | 6.24 (1.25) | 0.44 | 0.914 | 0.393 |
|  | **11** | 7.10 (0.98) | 6.72 (1.08) | 0.243 | 7.28 (1.24) | 6.61 (1.19) | 0.089 | 0.612 | 0.78 |
| **3-4** | **4** | 7.29 (0.58) | 7.81 (0.85) | 0.031^*^ | 8.34 (1.15) | 7.50 (1.28) | 0.035^*^ | 0.001^***^ | 0.373 |
|  | **6** | 7.52 (0.72) | 8.26 (1.15) | 0.019^*^ | 8.24 (1.00) | 7.31 (1.09) | 0.008^**^ | 0.013^*^ | 0.011^*^ |
|  | **8** | 7.84 (0.86) | 8.47 (1.15) | 0.057 | 8.20 (1.13) | 7.36 (1.15) | 0.024^*^ | 0.261 | 0.004^**^ |
|  | **11** | 8.22 (1.10) | 8.52 (1.18) | 0.408 | 8.36 (1.17) | 7.54 (1.37) | 0.048^*^ | 0.685 | 0.021^*^ |
| **4-5** | **4** | 7.69 (0.88) | 8.40 (1.67) | 0.102 | 8.08 (1.07) | 7.91 (1.35) | 0.67 | 0.217 | 0.314 |
|  | **6** | 7.98 (0.91) | 8.96 (1.82) | 0.04^*^ | 8.40 (1.07) | 8.20 (0.99) | 0.539 | 0.183 | 0.112 |
|  | **8** | 8.39 (1.02) | 9.07 (1.62) | 0.124 | 8.30 (1.28) | 8.41 (1.11) | 0.764 | 0.81 | 0.149 |
|  | **11** | 8.81 (1.22) | 9.31 (1.28) | 0.216 | 9.32 (1.20) | 8.48 (1.24) | 0.037^*^ | 0.194 | 0.045^*^ |
| **5-6** | **4** | 8.99 (1.03) | 10.19 (1.40) | 0.004^**^ | 8.92 (1.08) | 8.94 (1.08) | 0.949 | 0.829 | 0.003^**^ |
|  | **6** | 9.38 (1.17) | 10.51 (1.66) | 0.018^*^ | 9.40 (1.26) | 9.07 (1.09) | 0.386 | 0.958 | 0.003^**^ |
|  | **8** | 9.63 (1.39) | 10.81 (1.68) | 0.02^*^ | 9.53 (1.25) | 9.06 (1.25) | 0.24 | 0.815 | 0.001^***^ |
|  | **11** | 9.66 (1.19) | 10.92 (1.52) | 0.006^**^ | 9.53 (1.48) | 9.00 (1.24) | 0.224 | 0.764 | 0.001^***^ |
| **6-7** | **4** | 10.70 (1.07) | 11.51 (1.00) | 0.017^*^ | 10.59 (0.80) | 10.45 (1.30) | 0.678 | 0.733 | 0.006^**^ |
|  | **6** | 11.17 (1.17) | 12.22 (1.21) | 0.008^**^ | 11.12 (1.05) | 13.37 (1.62) | 0.401 | 0.9 | 0.665 |
|  | **8** | 11.76 (1.34) | 12.64 (1.52) | 0.06^*^ | 11.31 (1.49) | 10.37 (1.90) | 0.089 | 0.316 | 0.001^***^ |
|  | **11** | 11.29 (1.66) | 12.64 (2.01) | 0.027^*^ | 11.15 (2.03) | 10.17 (1.59) | 0.097 | 0.813 | 0.001^***^ |

*^*, **, ***:^ P-value*
