# Supplementary material for: Macro- and microstructural assessment of alveolar bone in adults with different vertical facial patterns using cone beam computed tomography
Source: Front Oral Health. 2026 Feb 16;7:1700017. doi: 10.3389/froh.2026.1700017 (PMC12950796; doi:10.3389/froh.2026.1700017)
Supplement: Supplementary file 3 [file Table3.docx]

Supplementary Table 3: Comparative statistical analysis of the maxillary trabecular bone measurements between male and female groups of normo- and hyper-divergent facial type using an independent t-test

| **Site** | **Variables** | **Males** | | | **Females** | | | ***P* value** | |
| --- | --- | --- | --- | --- | --- | --- | --- | --- | --- |
|  |  | **Normo-divergent**  **Mean (SD)** | **Hyper-divergent**  **Mean (SD)** | ***P* value** | **Normo-divergent**  **Mean (SD)** | **Hyper-divergent**  **Mean (SD)** | ***P* value** | **Normo-divergent**  **Male/Female** | **Hyper-divergent**  **Male/Female** |
| **1-2** | Tb Th mean (µm) | 1.66 (0.62) | 1.68 (0.40) | 0.723 | 1.85 (0.64) | 1.61 (0.71) | 0.275 | 0.361 | 0.971 |
|  | Tb SP mean (µm) | 1.83 (0.52) | 1.88 (0.54) | 0.770 | 2.07 (0.66) | 1.56 (0.61) | 0.017^*^ | 0.206 | 0.255 |
|  | Volume Ratio | 0.60 (0.58) | 0.49 (0.20) | 0.458 | 0.81 (0.63) | 0.60 (0.47) | 0.251 | 0.290 | 0.381 |
|  | Fractal Dimension | 2.10 (0.40) | 1.96 (0.56) | 0.370 | 1.76 (0.65) | 2.07 (0.27) | 0.057 | 0.053 | 0.444 |
| **2-3** | Tb Th mean (µm) | 1.98 (0.57) | 2.11 (0.44) | 0.072 | 1.76 (0.79) | 1.75 (0.81) | 0.972 | 0.514 | 0.034^*^ |
|  | Tb SP mean (µm) | 2.10 (0.66) | 2.14 (0.47) | 0.436 | 1.92 (0.63) | 1.82 (0.80) | 0.696 | 0.384 | 0.054 |
|  | Volume Ratio | 1.01 (1.10) | 0.69 (0.79) | 0.299 | 1.28 (0.89) | 0.72 (0.67) | 0.033^*^ | 0.406 | 0.904 |
|  | Fractal Dimension | 2.13 (0.57) | 1.95 (0.52) | 0.654 | 1.78 (0.82) | 1.99 (0.44) | 0.327 | 0.276 | 0.803 |
| **3-4** | Tb Th mean (µm) | 1.99 (0.80) | 2.26 (0.75) | 0.283 | 1.96 (0.71) | 1.66 (0.73) | 0.195 | 0.888 | 0.015^*^ |
|  | Tb SP mean (µm) | 2.04 (0.78) | 2.96 (0.93) | 0.002^**^ | 1.93 (0.79) | 1.74 (0.83) | 0.480 | 0.649 | 0.001^***^ |
|  | Volume Ratio | 0.59 (0.52) | 0.72 (1.00) | 0.602 | 1.29 (1.22) | 0.62 (0.53) | 0.031^*^ | 0.025^*^ | 0.678 |
|  | Fractal Dimension | 2.08 (0.47) | 1.97 (0.75) | 0.595 | 1.81(0.97) | 2.15 (0.35) | 0.154 | 0.280 | 0.345 |
| **4-5** | Tb Th mean (µm) | 2.19 (0.84) | 1.81 (0.76) | 0.138 | 1.73 (0.79) | 1.66 (0.70) | 0.597 | 0.082 | 0.836 |
|  | Tb SP mean (µm) | 2.12 (0.96) | 2.34 (1.22) | 0.622 | 2.28 (1.00) | 1.92 (0.68) | 0.200 | 0.606 | 0.722 |
|  | Volume Ratio | 0.78 (0.74) | 0.84 (1.15) | 0.850 | 1.19 (1.36) | 0.68 (0.62) | 0.140 | 0.257 | 0.578 |
|  | Fractal Dimension | 2.06 (0.42) | 2.31 (0.80) | 0.214 | 1.82 (0.92) | 2.41 (0.82) | 0.042^*^ | 0.309 | 0.726 |
| **5-6** | Tb Th mean (µm) | 1.78 (0.84) | 2.76 (1.17) | 0.004^**^ | 1.98 (0.86) | 1.95 (0.73) | 0.915 | 0.456 | 0.013^*^ |
|  | Tb SP mean (µm) | 1.51 (0.77) | 3.20 (1.79) | 0.001^***^ | 2.30 (1.02) | 2.36 (0.87) | 0.908 | 0.009^**^ | 0.044^*^ |
|  | Volume Ratio | 0.59 (0.45) | 0.81 (1.32) | 0.490 | 1.45 (1.67) | 0.66 (0.63) | 0.055 | 0.033^*^ | 0.640 |
|  | Fractal Dimension | 2.18 (0.38) | 2.09 (0.70) | 0.648 | 1.85 (0.72) | 2.26 (0.49) | 0.042^*^ | 0.084 | 0.382 |
| **6-7** | Tb Th mean (µm) | 2.08 (1.12) | 2.52 (1.32) | 0.262 | 2.13 (1.66) | 1.82 (0.70) | 0.451 | 0.913 | 0.043^*^ |
|  | Tb SP mean (µm) | 2.01 (0.75) | 3.09 (1.97) | 0.028^*^ | 1.80 (1.24) | 2.78 (1.61) | 0.038^*^ | 0.515 | 0.588 |
|  | Volume Ratio | 0.56 (0.39) | 0.68 (0.79) | 0.558 | 0.90 (0.83) | 0.49 (0.35) | 0.054 | 0.112 | 0.352 |
|  | Fractal Dimension | 2.12 (0.43) | 2.01 (0.62) | 0.534 | 2.22 (0.49) | 2.18 (0.40) | 0.813 | 0.509 | 0.313 |

*^*, **, ***:^ P-value*
