# Supplementary material for: Macro- and microstructural assessment of alveolar bone in adults with different vertical facial patterns using cone beam computed tomography
Source: Front Oral Health. 2026 Feb 16;7:1700017. doi: 10.3389/froh.2026.1700017 (PMC12950796; doi:10.3389/froh.2026.1700017)
Supplement: Supplementary file 4 [file Table4.docx]

Supplementary Table 4: Comparative statistical analysis of the mandibular trabecular bone measurements between male and female groups of normo- and hyper-divergent facial type using an independent t-test

| **Site** | **Variables** | **Males** | | | **Females** | | | ***P* value** | |
| --- | --- | --- | --- | --- | --- | --- | --- | --- | --- |
|  |  | **Normo-divergent**  **Mean (SD)** | **Hyper-divergent**  **Mean (SD)** | ***P* value** | **Normo-divergent**  **Mean (SD)** | **Hyper-divergent**  **Mean (SD)** | ***P* value** | **Normo-divergent**  **Male/Female** | **Hyper-divergent**  **Male/Female** |
| **1-2** | Tb Th mean (µm) | 1.07 (0.46) | 1.31 (0.51) | 0.135 | 1.51 (0.39) | 1.40 (0.53) | 0.462 | 0.002^**^ | 0.577 |
|  | Tb SP mean (µm) | 1.43 (0.65) | 1.82 (0.71) | 0.083 | 1.60 (0.67) | 1.67 (0.78) | 0.778 | 0.421 | 0.531 |
|  | Volume Ratio | 0.82 (0.80) | 0.38 (0.05) | 0.019^*^ | 0.61 (0.54) | 0.47 (0.20) | 0.314 | 0.327 | 0.056 |
|  | Fractal Dimension | 1.92 (0.41) | 2.00 (0.28) | 0.474 | 1.88 (0.40) | 1.94 (0.37) | 0.659 | 0.769 | 0.541 |
| **2-3** | Tb Th mean (µm) | 1.42 (0.76) | 1.36 (0.44) | 0.762 | 1.52 (0.79) | 1.31 (0.30) | 0.271 | 0.705 | 0.627 |
|  | Tb SP mean (µm) | 1.52 (0.81) | 1.64 (0.56) | 0.577 | 1.69 (0.93) | 1.45 (0.37) | 0.291 | 0.529 | 0.219 |
|  | Volume Ratio | 1.19 (1.16) | 0.44 (0.05) | 0.006^**^ | 0.60 (0.69) | 0.50 (0.43) | 0.601 | 0.057 | 0.526 |
|  | Fractal Dimension | 1.88 (0.59) | 2.07 (0.54) | 0.303 | 1.87 (0.53) | 1.89 (0.43) | 0.890 | 0.963 | 0.264 |
| **3-4** | Tb Th mean (µm) | 1.30 (0.53) | 2.22 (1.19) | 0.003^**^ | 1.79 (0.58) | 1.70 (0.82) | 0.691 | 0.009^**^ | 0.116 |
|  | Tb SP mean (µm) | 1.38 (0.68) | 1.90 (0.57) | 0.014^*^ | 2.10 (1.02) | 2.04 (1.31) | 0.891 | 0.013^*^ | 0.648 |
|  | Volume Ratio | 0.84 (0.77) | 0.56 (0.37) | 0.155 | 0.71 (0.88) | 0.53 (0.28) | 0.377 | 0.639 | 0.763 |
|  | Fractal Dimension | 1.92 (0.50) | 2.12 (0.260) | 0.130 | 1.89 (0.58) | 1.95 (0.43) | 0.724 | 0.855 | 0.145 |
| **4-5** | Tb Th mean (µm) | 1.78 (1.01) | 1.60 (0.47) | 0.460 | 1.60 (0.56) | 1.66 (0.61) | 0.739 | 0.474 | 0.722 |
|  | Tb SP mean (µm) | 1.55 (0.59) | 2.01 (0.64) | 0.026^*^ | 1.82 (0.74) | 1.89 (0.75) | 0.782 | 0.216 | 0.581 |
|  | Volume Ratio | 0.83 (0.73) | 0.55 (1.02) | 0.626 | 0.55 (0.42) | 0.51 (0.35) | 0.732 | 0.152 | 0.068 |
|  | Fractal Dimension | 2.07 (0.42) | 2.26 (0.31) | 0.105 | 2.13 (0.15) | 2.20 (0.35) | 0.424 | 0.547 | 0.551 |
| **5-6** | Tb Th mean (µm) | 1.89 (1.33) | 1.76 (0.89) | 0.720 | 1.76 (0.72) | 1.67 (0.64) | 0.682 | 0.709 | 0.725 |
|  | Tb SP mean (µm) | 2.47 (1.68) | 2.51 (1.53) | 0.612 | 1.91 (0.90) | 1.92 (0.74) | 0.968 | 0.195 | 0.447 |
|  | Volume Ratio | 0.74 (0.73) | 0.64 (0.65) | 0.739 | 0.64 (0.51) | 0.63 (0.89) | 0.699 | 0.609 | 0.807 |
|  | Fractal Dimension | 2.31 (0.75) | 2.14 (0.49) | 0.417 | 1.98 (0.63) | 2.10 (0.43) | 0.468 | 0.141 | 0.788 |
| **6-7** | Tb Th mean (µm) | 1.93 (0.58) | 1.97 (1.08) | 0.906 | 1.91 (0.88) | 1.73 (0.49) | 0.440 | 0.911 | 0.380 |
|  | Tb SP mean (µm) | 2.26 (1.84) | 2.45 (1.25) | 0.115 | 2.04 (0.78) | 2.17 (1.06) | 0.923 | 0.030^*^ | 0.629 |
|  | Volume Ratio | 0.49 (0.35) | 0.42 (0.57) | 0.489 | 0.60 (0.08) | 0.51 (0.31) | 0.242 | 0.373 | 0.539 |
|  | Fractal Dimension | 2.17 (0.14) | 2.09 (0.43) | 0.453 | 2.24 (0.14) | 2.16 (0.47) | 0.472 | 0.138 | 0.651 |

*^*, **, ***:^ P-value*
